# The validity, reliability and minimal clinically important difference of the patient specific functional scale in snake envenomation

**DOI:** 10.1371/journal.pone.0213077

**Published:** 2019-03-05

**Authors:** Charles J. Gerardo, Joao R. N. Vissoci, Leonardo P. de Oliveira, Victoria E. Anderson, Eugenia Quackenbush, Brandon Lewis, S. Rutherfoord Rose, Spencer Greene, Eric A. Toschlog, Nathan P. Charlton, Michael E. Mullins, Richard Schwartz, David Denning, Kapil Sharma, Kurt Kleinschmidt, Sean P. Bush, Nicklaus P. Brandehoff, Eric J. Lavonas

**Affiliations:** 1 Division of Emergency Medicine, Duke University School of Medicine, Durham, North Carolina, United States of America; 2 Duke Global Health Institute, Duke University, Durham, North Carolina, United States of America; 3 Department of Health and Biological Sciences, UniCesumar, Maringa, Paraná, Brazil; 4 CPC Clinical Research, Aurora, Colorado, United States of America; 5 Rocky Mountain Poison and Drug Center, Denver Health and Hospital Authority, Denver, Colorado, United States of America; 6 Department of Emergency Medicine, University of North Carolina, Chapel Hill, North Carolina, United States of America; 7 Health Science Center, Texas A&M University, College Station, Texas, United States of America; 8 Department of Emergency Medicine, Virginia Commonwealth University, Richmond, Virginia, United States of America; 9 Department of Emergency Medicine, Baylor College of Medicine, Houston, Texas, United States of America; 10 Department of Surgery, East Carolina University Brody School of Medicine, Greenville, North Carolina, United States of America; 11 Department of Emergency Medicine, University of Virginia, Charlottesville, Virginia, United States of America; 12 Division of Emergency Medicine, Washington University School of Medicine, St. Louis, Missouri, United States of America; 13 Department of Emergency Medicine and Hospital Services, Medical College of Georgia, Augusta, Georgia, United States of America; 14 Department of Surgery, Marshall Health, Huntington, West Virginia, United States of America; 15 Department of Emergency Medicine, University of Texas Southwestern Medical Center, Dallas, Texas, United States of America; 16 Department of Emergency Medicine, East Carolina University Brody School of Medicine, Greenville, North Carolina, United States of America; 17 Department of Emergency Medicine, University of California San Francisco Fresno, Fresno, California, United States of America; 18 Department of Emergency Medicine, Denver Health and Hospital Authority, Denver, Colorado, United States of America; Hong Kong Polytechnic University, HONG KONG

## Abstract

**Objective:**

Valid, reliable, and clinically relevant outcome measures are necessary in clinical studies of snake envenomation. The aim of this study was to evaluate the psychometric (validity and reliability) and clinimetric (minimal clinically important difference [MCID]) properties of the Patient-Specific Functional Scale (PSFS) in snakebite envenomation.

**Methods:**

We performed a secondary analysis of two existing snakebite trials that measured clinical outcomes using the PSFS as well as other quality of life and functional assessments. Data were collected at 3, 7, 10, and 17 days. Reliability was determined using Cronbach’s alpha for internal consistency and the intraclass correlation coefficient (ICC) for temporal stability at 10 and 17 days. Validity was assessed using concurrent validity correlating with the other assessments. The MCID was evaluated using the following criteria: (1) the distribution of stable patients according to both standard error of measurement (SEM) and responsiveness techniques, and (2) anchor-based methods to compare between individuals and to detect discriminant ability of a positive change with a receiver operator characteristic (ROC) curve and optimal cutoff point.

**Results:**

A total of 86 patients were evaluated in this study. The average PSFS scores were 5.37 (SD 3.23), 7.95 (SD 2.22), and 9.12 (SD 1.37) at 3, 7, and 10 days, respectively. Negligible floor effect was observed (maximum of 8% at 3 days); however, a ceiling effect was observed at 17 days (25%). The PSFS showed good reliability with an internal consistency of 0.91 (Cronbach’s alpha) (95% CI 0.88, 0.95) and a temporal stability of 0.83 (ICC) (95% CI 0.72, 0.89). The PSFS showed a strong positive correlation with quality of life and functional assessments. The MCID was approximately 1.0 for all methods.

**Conclusions:**

With an MCID of approximately 1 point, the PSFS is a valid and reliable tool to assess quality of life and functionality in patients with snake envenomation.

## Introduction

Snake envenomation is a common public health problem worldwide with annual estimates ranging from 421,000 to 1,842,000 cases per year, with 20,000 to 94,000 deaths per year.[1–3] The burden of disease is particularly pronounced in low- and middle-income countries, and has been reclassified as a neglected tropical disease by the World Health Organization, leading to further interest in the field.[2, 4, 5] Potential new therapies are being developed that will require clinical trials.[6–9] However, the existing literature lacks data regarding patient-centered outcome measures for use in snake envenomation clinical trials.[10] Consequently, prior trials have primarily used diagnosis-oriented endpoints, such as surrogate markers of coagulopathy, as primary outcomes.[11–15] Outcome measures that are patient centered and ideally patient reported will yield the most informative and clinically relevant information from the clinical trials evaluating potential snake envenomation therapies.[16]

The Patient-Specific Functional Scale (PSFS) is a widely used patient-reported outcome measure that identifies a short list of patient-chosen activities limited by the disease. The ability to perform these activities is rated by the patient and can be followed over time through repeat measurements.[17] This innovative approach has internal flexibility and measures only those activities considered important to the individual. Although primarily used in musculoskeletal disorders, this tool is not disease, organ system, or body part specific.[18–22] Additionally, the PSFS has the advantage of brevity (3 to 5 items) and validation in multiple languages such as English, Japanese, Finnish, Nepali and Portuguese.[23–26]

In 2012, a prospective observational study evaluated the number of potential outcome measures, including the PSFS, for assessing the recovery of limb function in snake envenomation.[27] That study showed good correlation with other assessment tools and responsiveness to change of the PSFS in snake envenomation, but further validity (such as construct, predictive and criterion) and reliability (such as internal consistency and temporal stability) need yet to be evaluated. Specifically, the minimally clinically important difference (MCID) was not assessed. The MCID indicates the minimal difference needed in a numeric scale that reflects a patient perception of change in a given health status. Defining the MCID is a key component in establishing the rigor of a patient-reported outcome intended to be used in patient-centered studies such as clinical trials. In 2015, a double-blind randomized clinical trial of snake envenomation used the PSFS as the primary outcome.[28] Data from the patients enrolled in these two studies are available to more fully assess the performance of the PSFS as an outcome measure of limb function recovery in snake envenomation. Therefore, the aim of this study was to evaluate the psychometric (validity and reliability) and clinimetric (MCID) of the PSFS in a snake envenomation population.

## Materials and methods

### Study design

We performed a *post-hoc* analysis of two multi-center, prospective studies conducted at 22 clinical sites across the southeastern United States (U.S.). The methods have been previously described in detail and are summarized below.[27,28] The first study used a prospective observational design (*Clinicaltrials*.*gov* NCT 01651299). All treatment provided, including the decision to administer or not administer antivenom, was at the discretion of the treating physician. The second study was a randomized, double-blinded, placebo-controlled trial, and all study treatments and assessments were performed in a blinded manner (*Clinicaltrials*.*gov* NCT 01864200). The patients were randomized in a concealed fashion to receive Crotalidae Polyvalent Immune Fab (Ovine) (CroFab) antivenom (FabAV) or placebo with the option of open-label rescue therapy in either treatment arm at the investigator’s discretion. All patients in both studies were enrolled in the emergency department, followed through their initial hospital encounter, and returned for outpatient assessments 3, 7, 14, 21, and 28 days after envenomation. Telephone assessments were performed between in-person assessments.

### Participants

Patients were eligible for inclusion if they were adolescents (12 years of age or older) or adults who were envenomated by a copperhead snake on an extremity, distal to the elbow or knee. Copperhead species was determined by investigator evaluation of snake, snake carcass, photographic of envenomating snake, captive snake, or patient identification of copperhead from a standard array of snake photographs. Although clinical evidence of venom effect (limb swelling and/or tenderness) was required, the venom effects did not need to be progressing at the time of enrollment. Patients must have presented for their initial episode of care within 24 hours of envenomation. Prisoners and pregnant or breastfeeding women were excluded. In addition, patients were excluded if they were unable to read and comprehend the consent document or written assessment tools or if they had a distracting injury or other condition that would limit the ability to make a reliable self-report of functionality status. Additionally, patients who sustained a previous snake envenomation to any body area or a previous injury to the envenomated limb within 30 days prior to enrollment were excluded. In the parent studies, written informed consent was obtained from all patients. Additionally, written parental assent was obtained for minors. Consent, assent, and assessment documents were available in English and Spanish. Spanish language documents were translated from English and back-translated from Spanish to ensure accuracy. The parent studies were approved by the Western Institutional Review Board and the institutional review boards of each institution [27,28].

### Instruments

#### Patient-Specific functional scale

The PSFS is a verbally administered three-item instrument. It evaluates a health condition’s impact on the ability to perform activities chosen by the patient. The patient is asked to identify “three important activities that you are unable to do or are having difficulty with as a result of your snakebite.” The patient rates for each item on an 11-point ordinal scale ranging from 0 (“unable to perform activity”) to 10 (“able to perform activity at the same level as before the injury or problem”). The patient re-rates the same three activities at each follow-up assessment. In this manner, the PSFS collects patient-oriented outcomes over time. An average of the three “important activity” scores was used for calculations. This instrument was first administered on the day 3 assessment.

#### Patient’s Global impression of change

The Patient’s Global Impression of Change (PGIC) is widely used to define clinically meaningful improvement in orthopedic, pain, and other studies. This instrument was first administered on day 3. This two-item assessment tool uses separate ordinal scales to assess change “since beginning treatment at this clinic” [29]. The first item was used for our analysis and is a 7-item Likert-type scale anchored at 1 (“no change or condition has worsened”). We considered patients with a score of 4 or less as unchanged. These PGIC responses range from “no change” to “somewhat better, but the change has not made any real difference.” Patients with a PGIC score of 5 or greater were considered to have perceived clinically important improvement. These responses range from “moderately better, and a slight but noticeable change” to “a great deal better, and a considerable improvement that has made all the difference.” This classification was used to determine patients’ perception of clinically relevant change to be used as an anchor for the clinimetric analysis of the PSFS. This approach has been used previously with the PSFS for other diseases.[30]

#### Functional assessments

Four different, well-established, functional outcomes measures were used to assess the external validity of the PSFS. We used the physical function element of the 36-Item Short Form Health Survey (SF-36) version 2 and the Patient-Reported Outcomes Measurement Information System Global-10 (PROMIS-10) to assess health-related quality of life (QoL) and physical function.[31,32] We used the Lower Extremity Functional Scale (LEFS) to measure lower extremity function.[33] We used the Disabilities of the Arm, Shoulder, and Hand (DASH) questionnaire to measure upper extremity function.[34] We correlated these functional outcomes with the PSFS score, and considered a positive moderate association as an indicator of good external validity. All functional assessments have shown good psychometric properties in prior literature [33–39].

The SF-36 is a measurement tool composed of a 36-item self-administered questionnaire with eight domain scales and two summary measures. Each item is an ordinal scale with 3–6 possible scores.[38, 40] We used the physical function domain for our analysis. The PROMIS-10 consists of 10 items that assess general domains of health and functioning, including overall physical health and overall quality of life. The LEFS questionnaire contains 20 questions about a person’s ability to perform everyday tasks. The LEFS can be used to evaluate the functional impairment of a patient with a disorder of one or both lower extremities. In addition, the LEFS can be used to monitor the patient over time and evaluate the effectiveness of an intervention.[33] The DASH is a 30-item self-administered questionnaire that evaluates upper extremity function. Each item is reported on a 5-point ordinal scale (1–5).[41] The LEFS and the DASH were correlated with the PSFS in patients with an upper or lower extremity injury.

### Data collection

Following screening and enrollment, each subject provided demographic information, past medical and medication history, and history of envenomation. In addition, a complete physical examination was performed. Data about the initial hospital encounter, including the date and time of arrival, the maximal extent of swelling, medication administration, laboratory test results, and adverse events, were recorded by study personnel at the time of care. Missing information was obtained from the hospital record. Formal study assessments were performed prior to discharge from the initial ED/hospital encounter and at 3, 7, 14, 21, and 28 days after envenomation.

### Analysis

Participant characteristics and the PSFS floor and ceiling effect are represented using descriptive statistics with frequency distribution, means, and standard deviations (SDs) when applicable. All analyses were conducted using the R Software for Statistical Computing program.

#### Psychometric property evaluation

We evaluated the reliability and external validity of the PSFS. Reliability was determined by assessing the internal consistency and temporal stability of the PSFS. Internal consistency was assessed using Cronbach’s alpha, with coefficients above 0.7 considered acceptable.[39] We assessed temporal stability using intraclass correlation (ICC) to determine the instrument’s variation in time.

External validity was assessed using the Spearman correlation of the PSFS measure with the SF-36 Health Outcomes Scale, the DASH, and the LEFS. A positive correlation (R > 0.60) was considered an indicator of external validity. Additionally, the change in the PSFS was correlated with the PGIC as an indicator of external validity. Higher variations in the PSFS were expected to correlate with higher scores of the PGIC.

#### Clinimetric property evaluation

To more definitively determine the MCID of the PSFS specifically for snake envenomation, we used two distribution-based methods and two anchor-based methods. For the first distribution-based method, the MCID was defined as the amount of variation in the PSFS score that must be observed before the change can be considered to exceed the standard error of measurement (SEM). The SEM was calculated using the formula (SD [1-r]^1/2^) where r is the ICC coefficient and the SD is the square root of the total variance. The SEM was multiplied by 1.65 to determine the 90% confidence interval (CI).[42] This value was multiplied by the square root of 2 to account for the errors introduced with repeated measurements.[43] For the second distribution-based method, the MCID was defined as the pooled SD of the difference between baseline and follow-up time multiplied by 0.5. This value corresponded to the amount of variation expected to identify a change in the PSFS score.[38] For the first anchor-based method, the MCID was defined as the smallest difference in the PSFS score that could predict the patients’ subjective perception of change.[44] This method calculated the MCID by identifying the optimal cut-off point (Youden’s Index) on the receiver operator characteristic (ROC) curve, which is considered to be the best discriminant value to distinguish improved from unchanged patients as defined by the PGIC.[42] Diagnostic performance (sensitivity, specificity, and area under the curve [AUC]) of the selected cut-off score were reported. For the second anchor-based method, the MCID was defined as the average of the difference in the PSFS between baseline and follow-up for the patients who reported subjective perception of improvement versus the patients who did not. [38] Once the MCID of the PSFS in snake envenomation was determined by the methods above, we externally validated the MCID by assessing the correlation of this value with measures of general health and functioning. Specifically, we compared the PROMIS-10, LEFS, DASH and SF-36 physical function at day 7 and 14 between patients with a change in PSFS of ≥ the MICD and < the MCID value.

## Results

Our sample was composed of 86 patients. The average age was 43.0 (SD 17.6) years, and the participants were mostly white (87%). Slightly more male patients (52%) participated in the study. Eight patients (9%) were adolescents. Most patients had lower extremity injuries (62%). When completing the PSFS, the main activities chosen by the participants were activities of daily living (e.g., carrying items, climbing stairs, providing self-care), sports and exercise (e.g., running, swimming, collective sports), and specific body movements (e.g., flexing the affected hand, moving a finger). Work-related activities represented only 11% of all activities chosen. (Table 1)

**Table 1:** Sociodemographic profile of the validation sample.

Demographics	
Age (years), Mean (SD)	43.0 (17.6)
Male, N (%)	39 (52)
Adolescent (%)	8 (9)
Race, N (%)	
White	65 (87)
Black	4 (5)
Asian	1 (1)
Other	4 (5)
Anatomic location, N (%)	
Lower extremity	46 (62)
Upper extremity	28 (38)
PSFS Content, N (%)	
Activities of daily living	79 (36)
Exercise, play games or sports	66 (30)
Body movement	30 (14)
Eat/Cook	17 (8)
Drive	16 (7)
Work-related	11 (5)

### Psychometric properties

The average PSFS score increased at each follow-up assessment. Negligible floor effect was observed (max of 8% at 3 days). However, a ceiling effect was observed after 7 days of treatment (Table 2). The reliability of the PSFS was adequate with good internal consistency (a Cronbach’s alpha of 0.91; 95% CI 0.88, 0.95) and good temporal stability (ICC value for the unchanged patients of 0.83; 95% CI 0.72, 0.89) (Table 2). External validity was demonstrated with a strong correlation of the PSFS scores with the SF-36 Physical Function scores, the PROMIS-10, and extremity functional assessments (Table 3).

**Table 2:** Psychometric properties of the PSFS.

	PSFS Scores		
	3 Days	7 Days	14 Days
Descriptive, Mean (SD)	2.86 (2.83)	5.39 (3.25)	7.89 (2.31)
Floor effect, N (%)	6 (8)	0 (-)	1 (0.01)
Ceiling effect, N (%)	9 (13)	18 (25)	8 (8.6)
**Reliability**			
Cronbach’s alpha (95% CI)	0.91 (0.88, 0.95)		
**Temporal stability**			
ICC (95% CI)	0.83 (0.72, 0.89)		

**Table 3:** Correlation of the PSFS score with quality of life and functionality measures.

	3 Days	7 Days	14 Days
**SF-36 Physical Function**	-	0.68**	0.72**
**PROMIS-10**	-	0.78**	0.83**
**LEFS**	0.83**	0.88**	0.86**
**DASH**	-0.44*	-0.67**	-0.82**

*p<0.01

** *p*<0.001

### Clinimetric properties

The two distribution-based methods yielded similar results. The MCID calculated using the SEM of the individual scale responses was 1.04. When using the pooled SD of the differences between the scale responses at baseline and follow-up, the MCID was 1.05.

The anchor-based methods showed similar MCID thresholds. At 3 days of follow-up, 39 (45%) patients were considered to have remained “unchanged” (scores of 4 or below on the PGIC), whereas 46 (53%) patients were considered to have improved (scores of 5 or greater on the PGIC). The optimal PSFS cut-off point to distinguish between unchanged and improved patients showed an MCID of 1.0 (Sensitivity = 0.60; 95% CI 0.52, 0.71, Specificity = 0.83; 95% CI 0.73, 0.89, AUC = 0.70; 95% CI 0.61, 0.73). Similarly, comparing the PSFS difference in unchanged versus improved patients on day 3 until day 14 showed an MCID of 0.98 as the threshold to detect differences in improvement across groups. (Table 4) As a measure of external validity of the PSFS MCID of 1.0, significantly higher scores in the SF-36 Physical Functioning, PROMIS-10 and LEFS were observed between participants with a PSFS change above and below 1.0 (Table 5).

**Table 4:** Clinimetric properties of the PSFS.

MCID Method	Value
Distribution-based^a^	
SEM	1.04
Pooled SD change from baseline	1.05
Anchor-based^b^^,^^c^	
Diagnostics MCID	1.00
Between-individual MCID	0.98

^a^For the distribution-based calculations, we used 3 and 7 days to see the closest difference in improvement after the event.

^B^For the AUC and diagnostics anchor-based MCID, we used 3 days as the metric to measure the first assessment post-hospitalization.

^c^For the between-individual and within-individual MCID, we used 3 and 14 days to compare the change from the start of treatment and full improvement.

**Table 5:** External validity assessment of the PSFS MCID.

	7 Days			14 Days		
Change in PSFS	≥1.0	<1.0	*p*	≥1.0	<1.0	*p*
**SF-36 PF**, Md (Q1;Q3)	70.0 (40.0;80.0)	42.5 (15.0;80.0)	0.16	87.5 (78.8;96.5)	55.0 (55.0;95.0)	0.04
**PROMIS-10**, Md (Q1;Q3)	43.2 (37.5;49.7)	37.1 (32.0;48.5)	0.06	50.1 (45.3;61.7)	42.6 (41.8;61.7)	0.11
**LEFS**, Md (Q1;Q3)	53.5 (32.3;65.8)	22.5 (17.5;34.8)	<0.001	67.0 (58.3;78.0)	52.0 (44.8;64.5)	0.04
**DASH**, Md (Q1;Q3)	35.8 (11.9;55.6)	20.0 (1.5;43.9)	0.21	12.9 (0.0;22.5)	2.5 (0.8;24.2)	0.82

## Discussion

The PSFS has been widely used with other clinical populations; however, to date, no previous study has evaluated the reliability, temporal stability, and MCID of the PSFS in patients with snake envenomation. Our results demonstrate that the PSFS is reliable, stable over time, externally valid, and strongly correlated with extremity functional assessment and other indicators of quality of life. The MCID was approximately 1.0 using multiple methodologies. This finding is important because the PSFS has been increasingly used as a patient-reported outcome measure due to its internal flexibility of content and ease of use.[45] These results inform the clinical significance of prior clinical trials and further refine a potential outcome measure for future snake envenomation studies.

The internal consistency of the PSFS in our study was excellent. This result is similar to the findings of studies performed in knee dysfunction, acute and chronic low back pain, cervical pain, chronic lateral epicondylitis, and chronic obstructive pulmonary disease (COPD). These studies found test-retest values ranging from 0.55–0.95.[18, 19, 46] In addition, the PSFS demonstrated stability over time, and these findings are similar to those in the existing literature, which show that the PSFS is consistent when measured at different times.[18] Additionally, our results show strong external validity when compared with other functional and quality of life instruments (SF-36, PROMIS-10, LEFS, and DASH) and prior studies.[22, 23, 47]

The PSFS has responsiveness and clinical relevance for patients in other disease groups.[18, 48] In addition, the PSFS has shown good reliability across items and time for the same groups of diseases and good between-group discrimination ability.[49] Other potential outcome measures of recovery of limb function from snake envenomation have not had this degree of evaluation, take more time to perform, and have other significant limitations. Other tools, such as the DASH and the LEFS, are limited by use in either the upper or lower extremity only, leaving a large proportion of snakebites unevaluable by a single tool.[33, 41] The measurement of swelling as an outcome measure does not demonstrate inter- and intrarater reliability, is not responsive to change during recovery, and lacks the advantages of a patient-reported outcome because this assessment does not measure recovery of function.[27, 50] Because of the limitations of other outcome measures, the strengths of the PSFS render it an excellent tool to assess the impact of venom-induced tissue injury in patients by measuring the meaningful outcome of recovery of function.

The MCID of the PSFS for a snakebite population was determined to be approximately 1 point. This finding was consistent across multiple distribution- and anchor-based methods. Moreover, we performed an external validation of an MCID of 1.0 with measures of general health and functioning. In this analysis, a change in PSFS of ≥ 1.0 (MCID) correlated with statistically significant higher PROMIS-10, SF-36 physical function, and LEFS scores compared to a change <1.0. The lack of association with the DASH may be related to its responsiveness, which has not been fully evaluated in snakebite. Although the MCID in chronic and spinal diseases has been found to be higher, similar values were found in other acute diseases involving the extremities.[19] This result provides additional assurance that an MCID of 1 point can used. When assessing the ability of the PSFS to determine the patient’s global impression of change, we found that an optimal MCID cut-off point of 1.0 on the ROC was highly specific (81%) for detecting the patient’s perception of change. However, the AUC was only 7.0 due to limited sensitivity in detecting change. Although a difference of 1 point in the PSFS indicated a clinically significant change between the groups, a lower PSFS value may lack the sensitivity in detecting an important difference between two groups even when one exists, which creates a risk of beta error when using this outcome measure. In future clinical trials, investigators may consider augmenting the PSFS with additional outcomes to provide further insight if a difference of less than 1.0 in the PSFS is found.

### Limitations

The results of this study should be taken within the context of the study’s limitations. The majority of the participants in this study were patients with mild copperhead envenomation. Because this type of envenomation is typically less severe than other snake envenomations, generalization to other pit viper species or to non—pit viper species should be performed carefully. In our study, no floor effects of the PSFS were detected, but this could potentially change in a more severely envenomed population. However, the PSFS has now been evaluated across a large spectrum of other non-envenomation disease states with similar findings, which should provide reassurance that this tool remains valid and reliable in snake envenomation cases that are more severe or from other species.

Because patients were undergoing emergent therapy for their snake envenomation upon enrollment, we were not able to determine the PSFS upon ED arrival or use that early time point in our analysis. The greatest improvement in the PSFS scores occurred during the early phase of recovery; therefore, a lack of data upon arrival likely resulted in an overestimation of the MCID in this study, indicating that the true MCID may be lower than we found. However, because our estimate is similar to other studies of extremity injuries, we suggest that an MCID of 1 point is a reasonable estimate.

Our evaluation of the PSFS is for recovery of limb function due to the tissue injury from the snake venom. Severe snake envenomation involves multiple organ systems or venom effect domains, which are not evaluated by the PSFS.[51] To fully assess outcomes in future snake envenomation clinical trials, the psychometric and clinimetric properties of other patient-oriented and/or patient-reported outcome measures for these clinical effects should be determined.

The PSFS functional activities chosen by each patient vary, which does not allow standard psychometric and clinimetric methods to be applied on the level of an individual activity. All of our analysis was performed on the aggregated PSFS score. The theoretical underpinning of this approach is that the PSFS is evaluating perception of functional impact by the snakebite regardless of the activity. This approach is consistent with the accepted evaluation of the PSFS in prior literature. [18–24]

## Conclusion

The PSFS is a valid, reliable tool in snake envenomation with good internal consistency, temporal stability, external validity, and correlation with other assessment tools, and this instrument is responsive to change. The PSFS should be considered an outcome measure of recovery of limb function due to tissue injury in future snake envenomation trials with an MCID of approximately 1 point.

## Supporting information

S1 Dataset PSFS Validation for snakebite data. (CSV)
